# AI-based pathomics in kidney diseases: progress and application

**DOI:** 10.1080/0886022X.2025.2598080

**Published:** 2025-12-16

**Authors:** Runmin Ding, Zexin Yang, Qiqin Yu, Junyi Zhou, Bin Ni, Ming Zheng, Zeping Gui, Min Gu, Zijie Wang

**Affiliations:** ^a^ Department of Urology, The Second Afffliated Hospital of Nanjing Medical University, Nanjing Medical University, Nanjing, Jiangsu, China; ^b^ The Second Clinical Medical College, Nanjing Medical University, Nanjing, Jiangsu, China; ^c^ Department of Urology, the First Affiliated Hospital of Nanjing Medical University, Nanjing, Jiangsu, China; ^d^ The First Clinical Medical College, Nanjing Medical University, Nanjing, Jiangsu, China

**Keywords:** Pathomics, artificial intelligence (AI), chronic kidney disease (CKD), renal fibrosis, kidney transplantation, renal cancer

## Abstract

In recent years, artificial intelligence (AI) has advanced significantly in the field of pathomics, enabling the transformation of pathological images into high-throughput, machine-readable datasets for quantitative analysis and precise diagnosis in renal pathology. AI-based pathomics has introduced innovative perspectives and sophisticated tools for detecting glomerular injury, evaluating renal interstitial fibrosis, monitoring transplanted kidney pathology longitudinally, and predicting outcomes in renal tumors. Despite its great promise, the application of AI-based pathomics in nephrology still faces several challenges, including complex data annotation, limited model interpretability, lack of comprehensive multi-modal data integration, and insufficient large-scale clinical validation. Future research should prioritize these challenges by enhancing multi-omics integration and promoting interdisciplinary collaboration, thereby advancing AI-based pathomics in nephrology and ultimately improving the precision and efficiency of patient care.

## 1. Introduction

With the rapid advancement of artificial intelligence (AI) technology, its integration into the medical field—particularly pathology—has brought transformative changes to traditional diagnostic practices. Pathomics involves extracting high-throughput, quantitative features from pathological images and converting them into datasets that support disease diagnosis, prognosis prediction, and treatment response assessment [1]. The application of pathomics not only enhances diagnostic accuracy and efficiency but also strengthens the foundation of precision medicine in disease treatment [2].

The pathological diagnosis of kidney diseases generally relies on histological examination [3]. However, traditional diagnostic methods face several limitations, such as subjectivity, limited reproducibility, and insufficient capacity to meet the demands of precision medicine. The emergence of AI offers a promising solution to these limitations [4]. By leveraging deep learning (DL) and machine learning (ML) algorithms, AI can autonomously detect and analyze subtle patterns and structures in pathological images, thereby facilitating faster and more accurate diagnoses of kidney diseases [5]. Furthermore, AI can extract quantitative features from pathological images, providing essential information for kidney disease prognosis [6].

In recent years, AI applications in kidney pathology have made significant progress. AI has shown great potential in various areas, including identifying kidney tissue structures, assessing lesion severity, and predicting treatment responses. For instance, DL models have been used to automatically identify key structures such as glomeruli and renal tubules and quantitatively assess lesions including glomerulosclerosis and tubulointerstitial fibrosis [7]. Additionally, AI technologies such as virtual staining are reducing dependence on traditional histochemical techniques, thereby enhancing both the efficiency and accuracy of pathological diagnosis [8]. These advancements underscore AI’s pivotal role in renal pathology and pave the way for innovative approaches to the precise diagnosis and treatment of kidney diseases. This review provides a comprehensive overview of the progress in AI-based pathomics in nephrology, with a focus on its applications in renal pathology image analysis, current challenges, and future research directions.

## 2. AI-based pathomics

### 2.1. Characteristics and standardized processing workflow of pathomics

**Figure 1.**
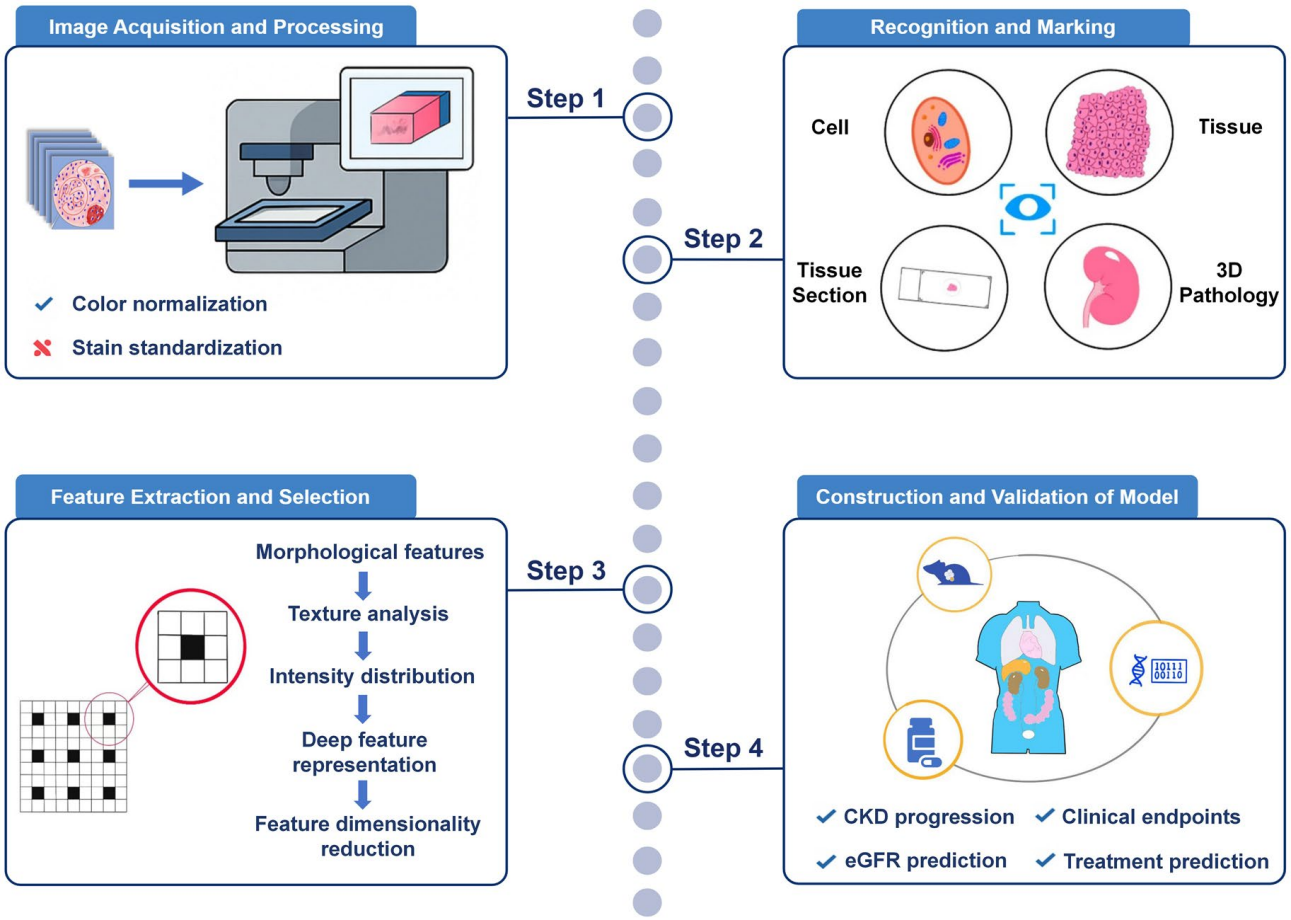
The standardized workflow of pathomics. The standardized workflow of pathomics includes four key steps: (i) image acquisition and processing; (ii) recognition and marking; (iii) feature selection and analysis; (iv) construction and validation of clinical model.

Pathomics, an emerging interdisciplinary field integrating AI and pathology, transforms pathological images into high-resolution, high-throughput datasets, offering novel approaches for disease diagnosis, prognosis evaluation, and treatment response prediction [9]. The standardized workflow for processing pathomics data typically involves several key steps (Figure 1). The first step is image acquisition and processing, where digital slide scanners convert tissue sections into whole-slide images (WSIs) [10]. These images undergo standardized preprocessing to mitigate staining inconsistencies, image noise, and other confounding factors [11]. The next step is feature extraction, where key pathological features—such as texture, morphology, and edge gradients—are derived from WSIs. These features are commonly categorized into low-level and high-level types [12]. Subsequently, feature selection and analysis are conducted using statistical and ML techniques to reduce dimensionality, select relevant features, and eliminate redundancy while preserving those crucial for disease diagnosis and prognosis [13]. Finally, model construction and validation are performed, where pathomics models are built using the selected features and evaluated for accuracy and generalizability *via* internal and external validation [14].

### 2.2. AI in clinical practice

**Figure 2.**
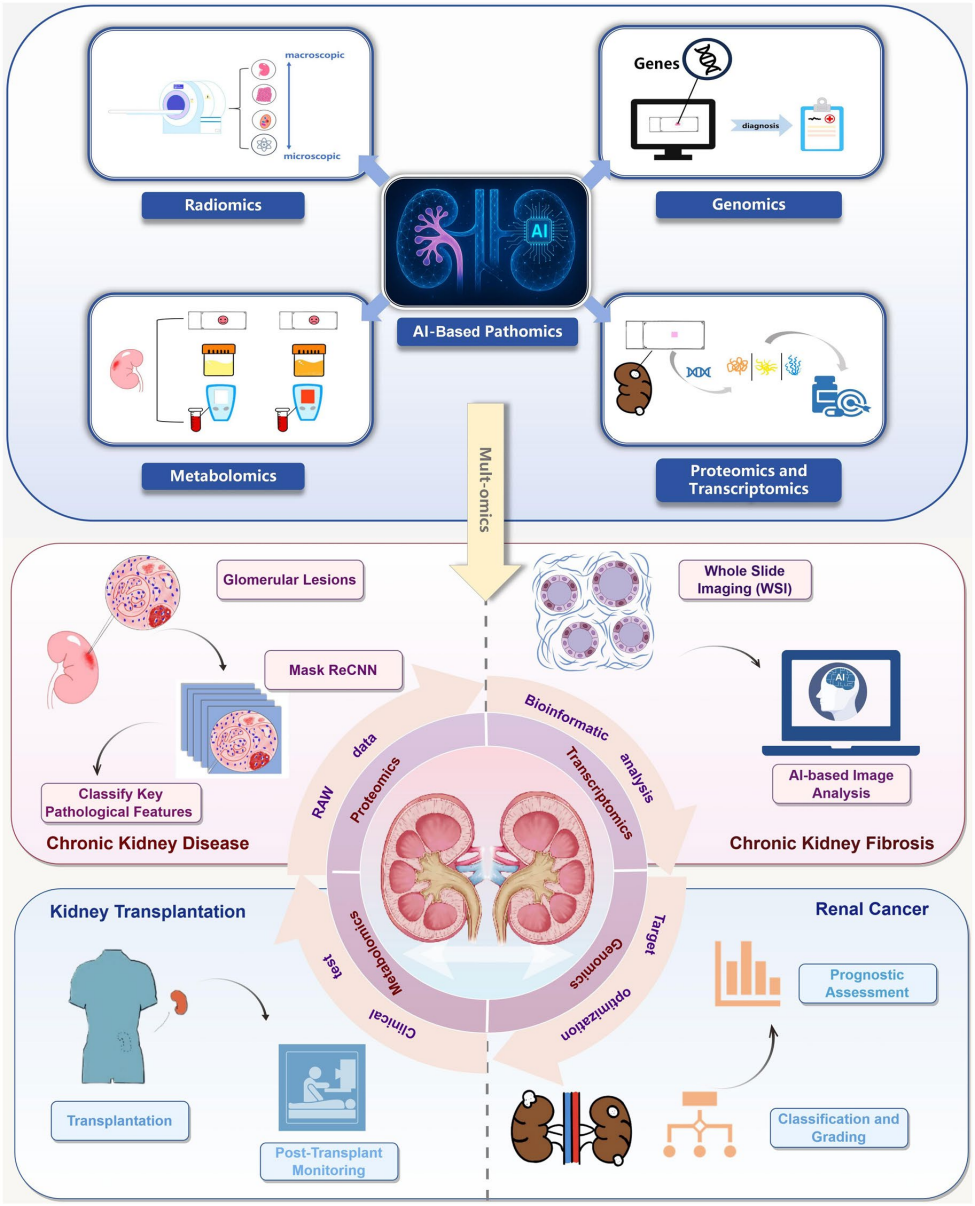
Applications of AI-based pathomics in kidney diseases. AI-based pathomics contributes to multiple aspects of kidney disease management: In glomerular diseases, it enables automatic quantification of glomerular morphology, facilitating pathological classification and prognosis assessment. In renal fibrosis, it supports fibrosis staging through image-based evaluation. In kidney transplantation, virtual biopsy systems powered by AI provide noninvasive assessment of graft quality and prediction of post-transplant lesions. In renal cancer, AI aids in malignancy detection, subtype classification, and prediction of tumor invasiveness based on integrated imaging and histopathological data. Additionally, multi-omics integration assists in elucidating molecular mechanisms, supporting precise diagnosis and targeted therapy.

AI has demonstrated significant impact across various fields, and clinical medicine is no exception. In ­particular, its application in the diagnosis and treatment of kidney diseases has been transformative. In medical imaging, deep learning-based algorithms can efficiently analyze multimodal data, enabling precise interpretation from macroscopic CT/MRI scans to microscopic pathological slides. For instance, a multi-staining fusion model developed by Sun Yat-sen University researchers allows prospective assessment of treatment response in lupus nephritis patients [15]. For chronic disease management, AI systems integrate diverse clinical indicators and social determinants of health (SDOH) to develop predictive models that outperform conventional approaches. Md Mohaimenul Islam’s team, for example, employed a random forest model incorporating SDOH to accurately predict chronic kidney disease risk in type 2 diabetic patients [16], offering insights for early intervention. Perhaps the most groundbreaking advancement lies in precision and personalized medicine. Kalluri Thishya et al. utilized artificial neural networks (ANN) and logistic regression models to predict tacrolimus bioavailability and post-transplant diabetes risk, respectively, enabling tailored therapeutic strategies [17]. Additionally, AI aids clinicians in decision-making and streamlines clinical data processing [18], enhancing both efficiency and accuracy in patient care. Despite these advances, several critical issues must be addressed before AI can be fully integrated into clinical practice. Limited diversity in training datasets may introduce algorithmic bias, potentially exacerbating healthcare disparities. Furthermore, if AI models are trained solely in simulated environments without real-world clinical validation, their diagnostic reliability may be compromised. Clinician expertise remains indispensable—AI should augment, not replace, medical judgment. Physicians must therefore develop a nuanced understanding of both clinical medicine and AI model logic to avoid misinterpretations. Finally, ethical and societal implications cannot be overlooked. Proactive measures are needed to ensure algorithms do not perpetuate systemic inequities, such as biases against underserved populations. Addressing these challenges will be crucial to harnessing AI’s full potential in nephrology and beyond, ultimately improving patient outcomes (Figure 2).

### 2.3. Application of AI in pathomics

AI has emerged as a transformative technology, reshaping diagnostic and therapeutic practices across the healthcare sector [19]. AI refers to computer algorithms designed to simulate human intelligence, enabling machines to perform tasks that typically require human cognition [20]. In medicine, AI primarily utilizes ML and DL algorithms to process large volumes of medical images and data, assisting healthcare professionals in disease diagnosis and treatment decision-making [21]. Convolutional neural networks (CNNs), a prominent type of DL model, are widely used for image recognition and analysis. CNNs use convolutional, pooling, and fully connected layers to automatically extract image features, facilitating classification and segmentation. In pathomics, CNNs are extensively used for the automated segmentation and classification of pathological images, significantly improving diagnostic accuracy [22]. With continuous advancements in AI, digital pathology has emerged as a game-changing technology. Digital pathology involves converting traditional tissue slides into WSIs using high-resolution scanners and applying computational technologies for efficient storage, management, and analysis [6]. This technology improves diagnostic efficiency and accuracy while providing foundational data critical to the advancement of pathomics [23]. The whole-slide imaging system is a core component of digital pathology [10]. The WSI system utilizes high-resolution scanners to generate digital images of pathological slides, facilitating the storage, management, and analysis of these images, thus laying the groundwork for the integration of AI into pathomics [24]. Moreover, virtual staining technology has effectively simulated the staining process in pathological images. By leveraging DL, AI simulates traditional staining effects, minimizing errors and reducing sample consumption. This enhances both diagnostic efficiency and image analysis accuracy [25,26].

### 2.4. Multi-Omics models in pathomics

Multi-omics integrates various omics technologies—including genomics, transcriptomics, proteomics, metabolomics, radiomics, and pathomics—to enable a comprehensive understanding of biological systems [13]. It facilitates the identification of molecular mechanisms, supporting clinical diagnosis and targeted therapy. Radiomics extracts high-throughput quantitative features from medical images to capture macroscopic tissue characteristics, whereas pathology focuses on microscopic structures. Integrating both enables multi-scale tissue analysis, thereby improving disease characterization [27]. Genomics connects gene expression patterns and mutations to pathological features, revealing underlying mechanisms, while proteomics characterizes protein expression and modifications to enhance disease understanding [28]. DL models further improve predictions of genetic mutations in kidney cancer using pathological features [14]. Proteomics analyzes protein expression and modifications to delineate tissue-specific proteomic profiles. The integration of proteomics and pathology enables correlations between pathological image features and protein expression data, offering insights into underlying proteomic mechanisms [29]. Multi-omics models offer improved diagnostic accuracy, predictive precision, and personalized treatment by integrating biological data across multiple layers [30,31]. They also assist in identifying novel mechanisms and therapeutic targets [32]. However, challenges persist in data integration due to inconsistencies among omics layers, necessitating advanced computational strategies [21,33]. High computational costs, limited interpretability, and inconsistent data standards across institutions hinder clinical translation and model generalizability, underscoring the need for further research to optimize multi-omics applications [34,35]. Furthermore, the Kidney Precision Medicine project (KPMP) innovatively integrates multi-omics and AI technologies to successfully construct a comprehensive renal atlas combining 3D pathological imaging with molecular profiling [36]. This breakthrough overcomes the limitations of conventional pathological diagnosis by enabling precise characterization spanning from macroscopic morphology to microscopic molecular features, thereby establishing a novel paradigm for disease classification, target discovery, and precision medicine in nephrology. The integration of multi-omics data presents multifaceted challenges, including inter-platform technical variability, limited algorithmic generalizability, inconsistent annotation standards, insufficient sample sizes, and the inherent tension between data privacy and sharing requirements – all of which constitute critical issues requiring resolution.

### 2.5. Explainable Artificial Intelligence in renal pathomics

Despite remarkable progress in AI-assisted renal pathology, the limited interpretability of AI models remains a major barrier to clinical translation. Explainable Artificial Intelligence (XAI) aims to bridge this gap by making the decision-making processes of complex models more transparent and understandable to clinicians. XAI techniques can be broadly categorized into feature attribution and visual explanation methods. For instance, SHapley Additive exPlanations (SHAP) provides both global and local insights into model predictions by quantifying each input feature’s contribution. In a recent study, Ghosh et al. employed SHAP to identify serum cystatin C (SCysC) and serum creatinine (SCr) as the most critical indicators for classifying chronic kidney disease (CKD) stages, while factors such as blood pressure and CRP levels were less influential. This enabled personalized predictions of disease progression and informed individualized treatment strategies [37]. In medical imaging, visual XAI methods such as Class Activation Mapping (CAM) and Gradient-weighted CAM (Grad-CAM) highlight specific image regions influencing model outputs. These techniques are highly valuable in kidney diagnostics—for example, by pinpointing tumor margins, fibrotic regions, or glomerular lesions in WSIs[38]. Integrating Grad-CAM into pathology workflows could reduce diagnostic variability and enhance trust among clinicians by offering spatially resolved justifications for AI decisions. Furthermore, combining XAI with multimodal data (e.g., genomics, radiomics, histopathology) may offer even greater insight into the biological rationale behind predictive models, enabling interpretable and mechanistically relevant AI systems in nephrology.

## 3. AI-based pathomics in kidney diseases

### 3.1. Chronic kidney disease

#### 3.1.1. Primary glomerular lesions

Chronic kidney disease (CKD) poses a significant global health burden, necessitating early and accurate diagnosis to prevent disease progression. Kidney biopsy remains the gold standard for diagnosis, primarily based on glomerular morphological evaluation [39]. AI-based pathomics has notably improved diagnostic accuracy and efficiency. Primary glomerular lesions are often caused by immune-mediated inflammation and are characterized by hypercellularity, sclerosis, and crescent formation [40]. Yang et al. developed a Mask ReCNN-LSTM model for glomerular detection and glomerulonephritis classification, achieving an accuracy of up to 94% [41]. Zeng et al. proposed a DL-based system that identified glomerular lesions and cell types with 92.2% accuracy, outperforming pathologists [42]. Chagas et al. integrated CNNs and support vector machines (SVMs) to classify glomerular proliferative lesions, achieving 100% accuracy in binary classification and 82% in multi-class settings [22]. These advancements highlight the transformative role of AI in glomerular lesion analysis, significantly enhancing diagnostic precision and efficiency.

AI has significantly enhanced the pathological assessment of IgA nephropathy. Ginley et al. developed a DL model that achieved 93% accuracy in distinguishing normal, partially sclerotic, and globally sclerotic glomeruli [43]. Uchino et al. trained an AI model on 15,888 glomerular images from 283 kidney biopsies, achieving AUCs of 0.986 for PAS staining and 0.983 for PAM staining in classifying key pathological features, with performance comparable to that of nephrologists [44]. Smerkous et al. developed a modified U-Net-based DL model for electron microscopy images, which automated segmentation of the podocyte-basement membrane interface and measurement of foot process width, reducing analysis time from hours to under one minute [45]. From glomerular detection and segmentation to lesion classification and intrinsic cell identification, AI models have not only improved diagnostic accuracy and efficiency but also provided pathologists with novel tools and insights. Despite methodological and application-related differences, these studies highlight the promise of AI in pathomics and lay a solid foundation for future research and clinical translation.

#### 3.1.2. Secondary glomerular lesions

Secondary glomerular lesions are typically caused by systemic diseases such as diabetic nephropathy (DN) and lupus nephritis (LN) [46]. These conditions often present with complex pathological features, making diagnosis more challenging. DN is among the most common microvascular complications of diabetes and is characterized by glomerular damage, including mesangial proliferation, Kimmelstiel-Wilson (K-W) lesions, and podocyte depletion [47]. Ginley et al. developed a DL model for identifying glomerular lesions in DN, achieving 93% accuracy in distinguishing normal, partially sclerotic, and globally sclerotic glomeruli [43]. Kitamura et al. demonstrated the diagnostic utility of AI in DN using immunofluorescence (IF) images. They collected IF images from 885 kidney biopsy cases and constructed a dataset comprising six types of IF images: IgG, IgA, IgM, C3, C1q, and fibrinogen. Using DL, they achieved 100% diagnostic accuracy for DN based solely on IF images [48]. Moreover, Lei et al. developed a CNN-based model to automatically evaluate key glomerular morphological characteristics in DN cases, such as the types of glomerular lesions and features within the glomerulus. Their findings demonstrated that the CNN model performed comparably to pathologists in predicting both baseline and long-term kidney function, including proteinuria and estimated glomerular filtration rate (eGFR) [49].

LN is a common renal complication of systemic lupus erythematosus (SLE) [50]. Zheng et al. developed a DL system to automatically detect and classify glomerular pathological alterations in kidney biopsy specimens from LN patients. The system accurately identified glomerular lesions—mild, severe, and sclerotic—with 95.1% accuracy, closely matching pathologists’ evaluations [51]. Cheng et al. further explored multi-staining DL models for predicting treatment response in LN. They trained a DL model on 880 digital slides from 245 patients, incorporating Hematoxylin and Eosin (H&E), Periodic Acid-Schiff (PAS), Periodic Acid-Methenamine Silver (PASM), and Masson’s Trichrome (TRI) staining techniques. The single-stain models achieved AUCs of 0.813, 0.841, 0.823, and 0.862, respectively. When all four staining models were integrated, the internal and external validation AUCs reached 0.901 and 0.840, respectively, outperforming conventional clinicopathological markers [15].

### 3.2. Chronic kidney fibrosis

Kidney fibrosis encompasses glomerulosclerosis and interstitial fibrosis, both of which are associated with renal cellular injury and inflammatory infiltration [52,53]. Intrinsic glomerular cells produce extracellular matrix, while inflammatory mediators released by injured glomerular and immune cells promote fibrosis progression, ultimately resulting in glomerulosclerosis [52]. Accurate identification and localization of glomeruli are essential for automated pathological diagnostics, facilitating precise disease assessment, prognosis prediction, and treatment planning. AI-based pathomics has emerged as a promising approach for standardizing fibrosis evaluation. Kolachalama et al. employed DL algorithms to systematically analyze kidney fibrosis biopsy images, accurately predicting CKD staging and renal survival at 1, 3, and 5 years. Furthermore, their findings demonstrated that the CNN model outperformed the pathologist-estimated fibrosis score (PEFS) in classifying fibrosis severity [54]. Bukowy et al. trained a CNN model on rat kidney tissue slides stained with Masson’s trichrome, achieving 80.2% accuracy when applied to human samples, despite interspecies variability [55]. Kannan et al. developed a DL model trained on 1,496 images to segment sclerotic glomeruli, reporting an F1 score of 0.632, though the model did not assess the proportion of sclerosis [56]. These studies underscore the potential of AI in automating glomerulosclerosis assessment, although challenges related to generalizability and segmentation accuracy persist.

Renal interstitial fibrosis is another key pathological feature in CKD progression, and its severity is strongly associated with patient prognosis [57]. Conventional assessments rely on visual estimation and semi-quantitative grading by pathologists; however, these methods often overlook subtle histological changes and suffer from considerable inter-observer variability. Recently, AI-based pathology has gained attention for its application in evaluating renal interstitial fibrosis. Zheng et al. developed a DL framework for predicting renal interstitial fibrosis and tubulointerstitial atrophy (IFTA) grades using Masson’s trichrome-stained WSIs from kidney biopsies. The framework integrates both local image patches and global WSI features, mimicking the pathologist’s diagnostic process and enabling accurate IFTA grading. The model achieved 71.8% accuracy on the test set and showed strong predictive performance for renal interstitial fibrosis [58].

### 3.3. Kidney transplantation

Kidney transplantation is a vital treatment for end-stage renal disease (ESRD); however, post-transplant immune responses may lead to pathological changes such as acute and chronic rejection, graft dysfunction, and fibrosis, which adversely affect long-term graft survival [59–61]. AI technologies hold promise for enhancing the accuracy and consistency of pathological assessments by automatically detecting and classifying transplant-related lesions from large-scale pathological image datasets. Augulis et al. applied DL models to pathological images of transplanted kidneys and successfully predicted clinical indicators, including CKD stage, serum creatinine levels, and graft survival, thereby enabling automatic identification and classification of pathological changes [62]. The extent of inflammatory infiltration in transplanted kidneys correlates with the severity of interstitial fibrosis and tubular atrophy. Hermsen et al. integrated multiplex tyramide signal amplification (mTSA) technology with DL models to analyze kidney transplant biopsy samples. Using high-density *in situ* labeling of cell surface markers, they identified inflammatory cell infiltration and assessed its association with fibrosis progression in renal grafts [63]. Moreover, accurate prediction of post-transplant renal function is essential for improving graft management. Yoo et al. developed a virtual biopsy system to objectively evaluate transplant kidney quality by predicting arterial sclerosis, arteriolar hyalinosis, interstitial fibrosis, tubular atrophy, and the proportion of sclerotic glomeruli, thereby supporting clinical decision-making regarding graft viability [64]. Luo et al. used CNNs to extract features from WSIs and combined them with clinical data from kidney transplant biopsies to construct a multimodal prediction framework [65]. Although research in this area remains limited and preliminary, these models show considerable promise for accurate identification of kidney graft fibrosis. They also hold promise for earlier detection and more reliable monitoring of disease progression in renal transplant patients.

### 3.4. Renal cancer

Renal cell carcinoma (RCC) is the most common type of kidney cancer, comprising subtypes such as clear cell RCC (ccRCC), papillary RCC (pRCC), and chromophobe RCC (cpRCC) [66,67]. AI has shown substantial progress in the classification and grading of RCC. Fenstermaker et al. developed a CNN that could distinguish normal renal tissue from malignant tumors and further classify them into ccRCC, pRCC, and cpRCC [68]. Additionally, Lu et al. proposed a CLAM-based DL model that classifies RCC subtypes using image-level labels and showed robust performance, even with smartphone microscope images [69]. The Fuhrman grading system, based on nucleolar morphology, remains a key prognostic tool in RCC evaluation. AI advancements have significantly improved the accuracy and consistency of Fuhrman grading in RCC. Kruk et al. utilized wavelet transformation for preprocessing and combined it with SVM and random forest classifiers to grade ccRCC based on the Fuhrman system [70]. Chanchal et al. developed RCCGNet, a CNN with shared residual blocks, capable of automatically grading RCC [71]. Furthermore, AI-based pathology has made significant strides in predicting RCC survival outcomes and treatment responses. For instance, Tian et al. developed a Lasso regression model to predict Fuhrman grading for ccRCC, subsequently integrating the Cox proportional hazards model to predict survival outcomes [72]. Similarly, Tabibu et al. proposed a CNN model for RCC subtype prediction, which was integrated with a Lasso-regularized Cox model to predict patient survival [73]. Moreover, AI technologies have shown promising capabilities in predicting gene mutations and expression directly from pathological images in RCC [74]. AI-based pathological genomics has achieved substantial progress in kidney tumor research, including tumor classification and grading, automated Fuhrman grading, prediction of survival and treatment responses, gene mutation and expression profiling, and analysis of tumor clonality and spatial heterogeneity. However, further validation and optimization are required to ensure the reliability and clinical applicability of these models across diverse datasets and settings.

## 4. Challenges in AI-based pathomics

While artificial intelligence has demonstrated its transformative potential in renal pathology, several technical challenges hinder its widespread adoption and integration into clinical practice. These challenges are multifaceted and require innovative solutions to fully realize AI’s potential in this field.

### 4.1. Challenges in image analysis and feature extraction

Several key challenges in AI-based renal pathology arise at the level of image processing, tissue segmentation, and feature extraction—processes that collectively define the accuracy and reliability of downstream predictions. One of the primary issues is staining variability, which occurs due to differences in tissue preparation and scanning protocols across institutions. These inconsistencies lead to variations in color and intensity, ultimately compromising model generalizability [75]. To address this, researchers have proposed normalization techniques as well as more recent generative adversarial network (GAN)-based stain transfer models. These approaches aim to standardize input data and improve consistency across multicenter datasets. Tissue segmentation presents additional complexities due to the heterogeneous and intricate nature of kidney microstructures. Accurate delineation of glomeruli, tubules, and fibrotic regions is often challenged by overlapping features and pathological distortion. Deep learning architectures such as U-Net and its variants have shown promise in addressing these challenges through their encoder-decoder structures, which enable both spatial localization and contextual understanding [76]. Equally important is feature extraction, which directly influences model learning. While traditional handcrafted features—based on morphology, texture, and intensity—have been used for decades, they often lack the ability to capture high-level histopathological context. Deep learning methods, particularly CNNs, enable hierarchical feature learning from raw image data. Transfer learning strategies allow fine-tuning of large pretrained models (e.g., ResNet, EfficientNet) on domain-specific datasets, significantly improving feature representation. Moreover, combining deep and handcrafted features into hybrid models has become a valuable strategy to balance accuracy with interpretability. Together, these technical aspects—preprocessing, segmentation, and feature extraction—form the foundation of AI-powered image analysis in renal pathology. Overcoming their limitations is essential for building robust and generalizable diagnostic models.

### 4.2. Challenges in model interpretability

A major obstacle to clinical adoption of AI in renal pathology is the limited interpretability of many deep learning models, which are often perceived as ‘black boxes’. In high-stakes clinical settings, it is essential that healthcare professionals understand the reasoning behind algorithmic decisions. Without transparency, clinicians may be reluctant to rely on AI outputs, regardless of performance metrics. To address this, the field has increasingly adopted XAI techniques. One widely used approach is SHAP, which quantifies the contribution of each input feature to the model’s output. Ghosh et al. successfully applied SHAP to interpret feature relevance in the prediction of CKD stages [37]. In the context of image analysis, techniques like Grad-CAM and CAM allow visualization of the specific regions in an image that influenced the model’s classification decision [77]. These tools help pathologists better understand how AI models interpret histological patterns and facilitate greater trust and acceptance in clinical workflows.

### 4.3. Challenges in data annotation and standardization

Another critical barrier lies in the quality and consistency of annotated training data. AI models rely heavily on labeled datasets, yet pathological interpretation often varies between experts, introducing noise and bias into the ground truth. Ginley et al. demonstrated that even state-of-the-art models exhibit inconsistencies in glomerulosclerosis classification when trained on subjectively annotated data [43]. Efforts to mitigate these challenges include the development of standardized annotation protocols and multi-expert consensus labeling. Collaborative initiatives across institutions are increasingly being pursued to build large-scale, high-quality datasets with consistent labels. However, these efforts are complicated by variations in scanner hardware, slide preparation, and imaging protocols. Therefore, data preprocessing and harmonization techniques—such as stain normalization and domain adaptation—remain essential to improving data quality across centers (Table 1).

**Table 1.** Glossary of key terms in AI-based pathomics.

Terms	Definition	Relevance to Pathomics
Artificial Intelligence	Computer algorithms designed to simulate human intelligence for tasks requiring cognition	Automates pathological image analysis
Pathomics	Quantitative transformation of pathological images into high-throughput, machine-readable datasets	Foundation of computational pathology
Deep Learning	Machine learning using multi-layered neural networks to automatically extract hierarchical features	Enables image segmentation and classification
Machine Learning	Algorithms that identify patterns and learn from data without explicit programming	Supports lesion detection and prediction tasks
Convolutional Neural Network	Deep learning architecture specialized for processing grid-like data using convolutional layers	Widely used for glomerular and tumor identification
Whole Slide Image	High-resolution digital scan of entire pathological tissue slides	Primary input for AI-based pathology analysis
Supervised Learning	Machine learning paradigm using labeled input-output pairs for training	Used for model training in diagnosis tasks
Unsupervised Learning	Machine learning approach that identifies hidden patterns in unlabeled data	Helps discover new tissue subtypes
Data Augmentation	Technique to artificially expand training datasets through image transformations	Improves training diversity and robustness
Stain Normalization	Computational standardization of color variations in histopathological stains	Enhances model generalization across centers
Multi-Omics Integration	Combined analysis of multiple biological data layers (genomics, proteomics, etc.)	Enables multi-scale disease understanding
Virtual Staining	AI-simulated histological staining from label-free tissue images	Reduces cost and preserves diagnostic quality
Feature Selection	Process of identifying and retaining the most informative features while reducing dimensionality	Improves model efficiency and prediction accuracy
Fuhrman Grading	Histopathological grading system for renal cell carcinoma based on nuclear features	AI assists in reproducibility of tumor grading
Radiomics	High-throughput extraction of quantitative features from medical imaging data	Complements pathomics for broader interpretation

**Abbreviations:** Artificial Intelligence (AI); Deep Learning (DL); Machine Learning (ML); Convolutional Neural Network (CNN); Whole Slide Images (WSI).

### 4.4. Ethical and regulatory challenges in clinical integration

Beyond technical limitations, growing ethical and regulatory concerns have become central to discussions on AI implementation in healthcare. The sensitive nature of renal pathology data—often involving high-resolution histopathological images, personal clinical metadata, and potentially genomic information—raises critical issues surrounding patient consent, data ownership, and cross-border data transfers. Ayorinde JOO et al. stressed that building trustworthy AI systems in nephropathology requires not only technical robustness but also rigorous attention to transparency, reproducibility, data governance, and contextual alignment between algorithm outputs and clinical decision-making workflows [78]. To address these multifaceted barriers, researchers have proposed strategies such as establishing standardized annotation protocols to reduce inter-observer variability and improve data consistency. There is also increasing emphasis on interpretable and auditable model architectures to enhance clinical trust. Notably, recent work has highlighted the promise of federated learning and other privacy-preserving techniques, which enable collaborative model development across institutions without transferring raw patient data [79]. These approaches collectively aim to ensure ethical compliance while promoting the safe and effective integration of AI into clinical workflows.

## 5. Conclusions and prospectives

Recent advancements in AI-based pathomics have profoundly influenced kidney disease research and clinical applications (Table 2). DL algorithms empower AI to automatically identify and analyze kidney structures—such as glomeruli, renal tubules, and interstitial regions—thus enabling the detection of glomerulosclerosis and proliferative lesions, assessment of fibrosis progression, and enhancement of diagnostic and prognostic accuracy. Virtual staining techniques, by reducing tissue damage and operational costs, further accelerate the advancement of pathological genomics.

**Table 2.** Applications of AI-based pathomics in kidney diseases.

Diseases	Models	Study Subjects and Sample Size	Model performance	Applications	Study Limitations	References
Primary Glomerular Lesions	CNN	653 Patients with kidney diseases	The glomerular disease classification model achieved a maximum accuracy of 0.94, while the glomerular lesion recognition model attained a highest area under the ROC curve of 0.947.	Glomerular disease classification and glomerular lesion classification	Single-Annotator Bias;Exclusion of Multimodal Data;Limited Disease and Lesion Coverage	Yang et al.(2022) [41]
	CNN	400 Patients with IgA nephropathy	The classification model for glomerular lesions achieved a Cohen’s kappa value of 0.912, indicating excellent agreement beyond chance. In contrast, the Cohen’s kappa for mesangial cell scoring was 0.420, suggesting moderate agreement.	Classification of glomerular lesions and evaluation of mesangial cell score	Limited to PAS Stains;Exclusion of Crescent Subtypes;Insufficient Endocapillary Hypercellularity Analysis	Zeng et al.(2020) [42]
	CNN	811 Renal WSIs	In the binary classification task, the accuracy of CNN-SVM reached 100%, and in the multi-class classification task, the average accuracy of CNN-SVM was 82%.	Classification of glomerular lesions: binary classification and multi-class classification	Limited Dataset Diversity;Exclusion of Other Staining Methods;Narrow Focus on Hypercellularity	Chagas et al.(2020) [22]
	CNN	283 Patients with kidney disease	It helps pathologists increase the sensitivity of classification by 0.09 and the specificity by 0.04.	Classification of Glomerular Lesions	Annotator Variability;Limited Dataset Scope;Clinical Context Exclusion	Uchino et al.(2020) [44]
	CNN	820 EM images of renal biopsies	The measurement time of FPW was reduced from 6 to 8 hours for expert technicians to approximately 45 seconds per biopsy image.	Evaluate the degree of podocyte damage	Limited Dataset Scope;EM Image Storage Requirements;Generalizability Constraints	Smerkous et al.(2024) [45]
Secondary Glomerular Lesions	CNN and RNN	54 Human WSIs and 25 mouse WSIs	The Cohen’s kappa value of the glomerular pathological grading model is 0.55.	Pathologically grade the glomeruli of diabetic nephropathy	Limited Clinical Validation;Exclusion of Tubulointerstitial Compartment;Stain Dependency	Ginley et al. (2019) [43]
	CNN	885 Patients with diabetic nephropathy	The area under the ROC curve of the immunofluorescence image diagnosis model ranges from 0.93 to 1.00.	Diagnose diabetic nephropathy through immunofluorescence images	Limited Dataset Scope;Exclusion of Light Microscopy Comparison;Single-Center Data Source;	Kitamura et al.(2020) [48]
	CNN	Renal biopsy samples of 631 patients with diabetic nephropathy	The Cohen’s kappa value of the automatic glomerular lesion classification model is 0.624, indicating a moderate consistency with the classification results of pathologists.	Automatically classify glomerular lesions and utomatically quantify the morphological characteristics of glomeruli	Limited Glomerular Analysis Scope;Single-Center Validation Constraint;Absence of Multi-Staining Verification	Lei et al.(2024) [49]
	CNN	163 Patients with LN	The accuracy rate of evaluating the degree of lesions is 0.807, and the κ value of the model is 0.855.	Evaluate the degree of glomerular lesions in lupus nephritis	Single-Stain Analysis Limitation;Exclusion of Tubulointerstitial Features;Lack of Multicenter Validation	Zheng, Zhang, et al.(2021) [51]
	LNCR-DL	880 Digital slides from 245 patients	The predictive performance of the LNCR-DL model is significantly better than that of traditional clinicopathological parameters.	Predict the treatment response of patients with lupus nephritis 12 months after receiving induction therapy	Limited Treatment Regimen Scope;Retrospective Study Design;Absence of Prospective Validation	Cheng et al.(2025) [15]
Chronic Kidney Fibrosis	CNN	171 CKD patients	The model outperforms pathologists in predicting the stage of CKD, serum creatinine levels, proteinuria, as well as the renal survival rates at 1 year, 3 years, and 5 years.	Predict the stage of CKD and renal survival rate; evaluate the degree of renal fibrosis	Single-Stain Analysis Constraint;Retrospective Study Design Limitation;Lack of Treatment Effect Integration	Kolachalama et al. (2018) [54]
	CNN	87 Slices of rat renal tissue and 6 slices of human renal tissue	The precision of the glomerular lesion recognition model in identifying glomeruli in human renal tissue is 80.2%.	Identify and localize the glomeruli	Limited Human Tissue Training Data;Absence of Automated Injury Scoring Integration;Medullary Region Detection Challenges	Bukowy et al.(2018) [55]
	CNN	171 CKD patients	The accuracy rate of the glomerulosclerosis classification model is 92.67%.	Classification of glomerulosclerosis: global sclerosis and non-global sclerosis	Small Training Dataset Size for Deep Learning;Absence of Multi-Label or Multimodal Feature Learning;Lack of External Validation from Other Institutions;No Quantitative Analysis for NPS Glomeruli Segmentation;Absence of Clinical-Outcome Correlation and Prognostic Evaluation	Kannan et al.(2019) [56]
	Glpathnet	95 WSIs	The κ value of the model for assessing the IFTA grade is 0.622.	Assess the IFTA grade	Interobserver Variability in Fibrosis Grading;Limited External Validation Across Institutions;Single-Stain Input Constraint (Trichrome Only);Lack of Integration with Clinical Parameters (e.g., eGFR, Creatinine);	Zheng, Cassol, et al. (2021) [58]
Kidney transplantation	CNN	1021 WSIs	Quantify the acute and chronic components of tubulointerstitial injury through multivariate analysis. The model is reproducible across different datasets and is associated with Banff scores and renal function indicators.	Evaluate the acute and chronic injuries in biopsies of renal transplants	Limited External Validation Scope;Semiquantitative Banff Score Dependency;Absence of Longitudinal Outcome Correlation	Augulis et al.(2024) [62]
	CNN and mTSA	Renal biopsy samples from 22 patients with DGF 6 weeks after renal transplantation	The F1 value of the detection model for the degree of inflammatory cell infiltration in transplanted kidneys is between 0.77 and 0.82.	Quantify the degree of inflammatory cell infiltration in transplanted kidneys	Limited Sample Size;Single-Center Retrospective Design;Technical Constraints of mTSA Staining	Hermsen et al.(2021) [63]
	CNN	219 Transplant patients	The accuracy of predicting the estimated glomerular filtration rate and the decline of renal function after transplantation has increased by 0.14 and 0.12 respectively.	Predict the estimated glomerular filtration rate of the kidney and renal function after kidney transplantation	Limited Sample Size and Retrospective Design;Single-Center Study Without External Validation;Exclusion of Frozen Biopsies and Time-Intensive FFPE Processing	Luo et al.(2021) [65]
	TRIPOD	14,032 Kidney allograft biopsies	The multi-AUC of the model for predicting IFTA lesions is 0.830, and the AUROC reaches 0.900. For the model in the Columbia University cohort, the multi-AUC for IFTA is 0.723, and the AUROC is 0.905. In the model of the cohort from Sun Yat-sen University, the multi-AUC for IFTA is 0.798, and the AUROC is 0.935.	Evaluate organ quality, optimize allograft allocation, and distinguish between donor-derived and acquired lesions after transplantation	Interobserver Variability in Biopsy Evaluation;Heterogeneity in Biopsy Techniques Across Centers;Limited Predictors Beyond Routine Donor Parameters	Yoo et al.(2024) [64]
Renal Cancer	CNN	42 RCC patients and 800 normal parenchyma samples	The overall accuracy of the model for identifying RCC in distinguishing between normal renal parenchyma and RCC is as high as 99.1%. The sensitivity for identifying RCC is 100%, and the specificity is 97.1%.	Identify the presence of renal cell carcinoma; Distinguish the histological subtypes of renal cell carcinoma; Predict the tumor grade of renal cell carcinoma	Limited Patient Sample Size;Lack of Renal Mass Biopsy Validation;Dependence on Digital Pathology Infrastructure	Fenstermaker et al. (2020) [68]
	CLAM	Public RCC WSI dataset: 884 WSIs; Independent BWH RCC WSI dataset: 135 WSIs	The average area under the test curve of the classification model for renal cell carcinoma subtypes reaches 0.991 ± 0.004.	Classification of renal cell carcinoma subtypes; Detection of lymph node metastasis	Limited Training Data Size;Dependence on Slide-Level Labels;Computational Resource Intensity	Lu et al. (2021) [69]
	SVM and RF	70 RCC patients	The average accuracy of the overall classification model reaches 96.7%. The recognition accuracy for Fuhrman grade 1 is 0.981, for grade 2 is 0.976, for grade 3 is 0.943, and for grade 4 is 0.874.	Assist in pathological diagnosis;Improve the diagnostic efficiency;Provide visualized results	Limited Dataset Size;Lack of External Validation;Computational Complexity	Kruk et al.(2017) [70]
	RCCGNet	3,442 Image patches in the training set	On the KMC kidney dataset, the accuracy of the RCCGNet classification model reaches 90.14%; on the BreakaHis dataset, the accuracy of the RCCGNet classification model is 90.09%.	Assist in the pathological diagnosis of renal cell carcinoma;Universally applicable for the analysis of histopathological images of multiple organs	Limited Dataset Diversity;Absence of External Validation;Computational Resource Constraints	Chanchal et al.(2023) [71]
	Lasso	395 ccRCC patients	The average ROC AUC of the model is 0.84.	Perform grading judgment on cases; conduct correlation analysis with overall survival rate	Limited Generalizability to Other Grading Systems;Dependence on Manual ROI Selection;Suboptimal Nuclei Segmentation in Challenging Cases	Tian et al.(2019) [72]
	CNN,DAG-SVM and Lasso-regularized COX	1,027 Tumor slice images of ccRCC, 303 tumor slice images of pRCC, and 254 tumor slice images of cpRCC	The best value of the AUC of the model for distinguishing RCC from normal tissues can reach 0.98, and it is 0.95 for cpRCC. The Kappa score of the model for distinguishing RCC subtypes is 0.794. The model for predicting the survival outcome of ccRCC has a significant correlation with the survival outcome of patients.	Classification of RCC and normal tissues as well as RCC subtypes; Prediction of survival outcomes	Limited Data Availability for Rare Subtypes;Class Imbalance in Multi-Class Classification;Computational Challenges with Whole-Slide Images	Tabibu et al.(2019) [73]

**Abbreviations:** Convolutional Neural Network (CNN); Support Vector Machines (SVM); Transparent Reporting of a Multivariable Prediction Model for Individual Prognosis or Diagnosis (TRIPOD); Whole Slide Images (WSI); Electron microscopy (EM); Lupus nephritis(LN); Diabetic nephropathy (DN); Chronic kidney disease (CKD); Delayed graft function (DGF); Renal cell carcinoma (RCC); Clear cell renal cell carcinoma (ccRCC); Papillary renal cell carcinoma (pRCC); Chromophobe renal cell carcinoma (cpRCC).

However, several challenges persist, including the need for large, high-quality annotated datasets, standardization protocols, and stringent quality control measures, all of which hinder the generalizability of AI models. The low interpretability of AI decision-making further impedes its clinical integration. In addition, many current studies emphasize single-staining techniques or isolated features, lacking the multi-modal and multi-scale integration necessary for comprehensive analysis. The lack of large-scale, multi-center clinical validation underscores the necessity for robust real-world evaluations of AI-based applications in renal pathology.

In conclusion, while AI-based pathomics has demonstrated considerable potential in revolutionizing renal disease diagnostics and therapeutic monitoring, three fundamental challenges must be resolved to facilitate clinical implementation. Primarily, constraints in data acquisition and annotation quality may be addressed through federated learning architectures [80], which facilitate secure, privacy-preserving collaborative model development across institutions while circumventing data-sharing barriers. Secondly, standardization challenges encompassing both technical (e.g., staining variability) and operational (e.g., scanning protocols) aspects could be ameliorated through the establishment of consensus guidelines for digital pathology workflows coupled with advanced computational stain normalization techniques. Most critically, enhancing model interpretability—an essential prerequisite for clinical adoption—can be achieved through the integration of explainable AI methodologies, particularly attention-based mechanisms that provide spatially resolved feature importance maps, as evidenced by their successful deployment in nephropathological image analysis. Future developmental efforts should prioritize the creation of regulatory-compliant, clinically validated AI systems that holistically incorporate these solutions to bridge the translational gap.

## Data Availability

No data were generated or analyzed in the current research.
